# Amputation of the Entire Scapulum

**Published:** 1844-10-19

**Authors:** 


#### AMPUTATION OF THE ENTIRE SCAPULUM.

M. Rigaud, Professor of Clinical Surgery at the Faculty of Strasburgh, forwarded to the academy the model of a scapulum which he had taken away, along with a portion of the clavicle, from a man, fifty-one years of age. The patient, a grenadier of the Imperial Guard, bore, in 1841, a tumour of the superior portion of the left arm, which necessitated amputation at the shoulder-joint, an operation which M. Rigaud performed. The wounds resulting from the operation healed, and the patient was well during eight months. At that epoch an osseous tumour formed in the axillary region, evidently proceeding from the anterior angle of the scapulum. M. Rigaud thought it necessary to take out the entire scapulum along with the external extremity of the clavicula. This laborious operation was performed successfully in 1842; the patient recovered in the course of two months, and has ever since enjoyed good health.—*Ibid.*

